# Supplementary material for: Public attitudes toward allocating scarce resources in the COVID-19 pandemic
Source: PLoS One. 2020 Nov 4;15(11):e0240651. doi: 10.1371/journal.pone.0240651 (PMC7641460; doi:10.1371/journal.pone.0240651)
Supplement: S1 File — (PDF) [file pone.0240651.s001.pdf]

Public Attitudes Toward Allocating Scarce Resources in the Covid-19 Pandemic

Sample Demographics

Table 1. Demographic characteristics of the sample in Experiment 1

Demographic		Frequency	Percent
Self-identified Biological Sex	Female	226	36.30
Age		37 (12)	
Race	White	438	70.40
	Black	66	10.60
	Hispanic	47	7.60
	Asian	63	10.10
	Other	7	1.10
Native Language	English	588	95.00
Education	High School	56	9.00
	Some College	97	15.60
	2 Year Degree	53	8.50
	4 Year Degree	298	47.90
	Postgraduate/Professional	117	18.80
Hospital Visit in 5 Years	Yes	224	36.00
Hospital Training	Yes	134	21.90

Table 2. Demographic characteristics of the sample in Experiment 2

Demographic		Frequency	Percent
Self-identified Biological Sex	Female	274	45.10
Age		38 (35)	
Race	White	429	70.70
	Black	52	8.60
	Hispanic	39	6.40
	Asian	79	13.00
	Other	6	1.00
Native Language	English	564	92.90
Education	High School	55	9.10
	Some College	103	17.00
	2 Year Degree	54	8.90
	4 Year Degree	282	46.50
	Postgraduate/Professional	111	18.30
Hospital Visit in 5 Years	Yes	218	35.90
Hospital Training	Yes	117	19.30

Table 3. Demographic characteristics of the sample in Experiment 3

Demographic		Frequency	Percent
Self-identified Biological Sex	Female	246	38.50
Age		39(13)	
Race	White	501	78.40
	Black	73	11.40
	Hispanic	23	3.60
	Asian	34	5.30
	Other	8	1.30
Native Language	English	602	94.20
Education	High School	48	7.50
	Some College	78	12.20
	2 Year Degree	42	6.60
	4 Year Degree	349	54.60
	Postgraduate/Professional	122	19.10
Hospital Visit in 5 Years	Yes	241	37.70
Hospital Training	Yes	165	25.80
