# Supplementary material for: Public attitudes toward allocating scarce resources in the COVID-19 pandemic
Source: PLoS One. 2020 Nov 4;15(11):e0240651. doi: 10.1371/journal.pone.0240651 (PMC7641460; doi:10.1371/journal.pone.0240651)
Supplement: S2 File — (PDF) [file pone.0240651.s002.pdf]

Public Attitudes Toward Allocating Scarce Resources in the Covid-19 Pandemic

Detailed Survey Materials

Experiment 1 [Fscare: plentiful=0, scarce=1; Ftype: 0=order, 1=serious, 2=lives, 3=important]

[Plentiful – Order] A pandemic is impacting many countries. People infected could die without immediate medical care. The amount of infected people is growing and might soon exceed the capacity of hospitals. The triage team at County General Hospital is responsible for deciding the order that new patients receive lifesaving resources, such as ventilators or ICU beds. The team has recently instituted a new policy. According to this policy, patients will receive lifesaving resources in the order in which they arrive, with those arriving to the hospital first being prioritized. County General is very well supplied, so there are many more lifesaving resources available than there are patients. Because of this, no patients will go without lifesaving resources who need them.

[Plentiful – Serious] A pandemic is impacting many countries. People infected could die without immediate medical care. The amount of infected people is growing and might soon exceed the capacity of hospitals. The triage team at County General Hospital is responsible for deciding the order that new patients receive lifesaving resources, such as ventilators or ICU beds. The team has recently instituted a new policy. According to this policy, patients will receive lifesaving resources in the order of the seriousness of illness, with those who are the worst off being prioritized. County General is very well supplied, so there are many more lifesaving resources available than there are patients. Because of this, no patients will go without lifesaving resources who need them.

[Plentiful – Lives] A pandemic is impacting many countries. People infected could die without immediate medical care. The amount of infected people is growing and might soon exceed the capacity of hospitals. The triage team at County General Hospital is responsible for deciding the order that new patients receive lifesaving resources, such as ventilators or ICU beds. The team has recently instituted a new policy. According to this policy, patients will receive lifesaving resources in the order that saves the most lives, with those having the best chances of recovery being prioritized. County General is very well supplied, so there are many more lifesaving resources available than there are patients. Because of this, no patients will go without lifesaving resources who need them.

[Plentiful – Important] A pandemic is impacting many countries. People infected could die without immediate medical care. The amount of infected people is growing and might soon exceed the capacity of hospitals. The triage team at County General Hospital is responsible for deciding the order that new patients receive lifesaving resources, such as ventilators or ICU beds. The team has recently instituted a new policy. According to this policy, patients will receive lifesaving resources in the order of their importance, with those standing to contribute the most to society being prioritized. County General is very well supplied, so there are many

more lifesaving resources available than there are patients. Because of this, no patients will go without lifesaving resources who need them.

[Scarce – Order] A pandemic is impacting many countries. People infected could die without immediate medical care. The amount of infected people is growing and might soon exceed the capacity of hospitals. The triage team at County General Hospital is responsible for deciding the order that new patients receive lifesaving resources, such as ventilators or ICU beds. The team has recently instituted a new policy. According to this policy, patients will receive lifesaving resources in the order in which they arrive, with those arriving to the hospital first being prioritized. County General is not well supplied, so there are many more patients than there are lifesaving resources available. Because of this, many patients will go without lifesaving treatment who need them.

[Scarce – Serious] A pandemic is impacting many countries. People infected could die without immediate medical care. The amount of infected people is growing and might soon exceed the capacity of hospitals. The triage team at County General Hospital is responsible for deciding the order that new patients receive lifesaving resources, such as ventilators or ICU beds. The team has recently instituted a new policy. According to this policy, patients will receive lifesaving resources in the order of the seriousness of illness, with those who are the worst off being prioritized. County General is not well supplied, so there are many more patients than there are lifesaving resources available. Because of this, many patients will go without lifesaving treatment who need them.

[Scarce – Lives]
A pandemic is impacting many countries. People infected could die without immediate medical care. The amount of infected people is growing and might soon exceed the capacity of hospitals. The triage team at County General Hospital is responsible for deciding the order that new patients receive lifesaving resources, such as ventilators or ICU beds. The team has recently instituted a new policy. According to this policy, patients will receive lifesaving resources in the order that saves the most lives, with those having the best chances of recovery being prioritized. County General is not well supplied, so there are many more patients than there are lifesaving resources available. Because of this, many patients will go without lifesaving treatment who need them.

[Scarce – Important] A pandemic is impacting many countries. People infected could die without immediate medical care. The amount of infected people is growing and might soon exceed the capacity of hospitals. The triage team at County General Hospital is responsible for deciding the order that new patients receive lifesaving resources, such as ventilators or ICU beds. The team has recently instituted a new policy. According to this policy, patients will receive lifesaving resources in the order of their importance, with those standing to contribute the most to society being prioritized. County General is not well supplied, so there are many more patients than there are lifesaving resources available. Because of this, many patients will go without lifesaving treatment who need them.

[CC1] There are enough lifesaving resources for every patient who needs them.

- Yes
- No

[CC2] Lifesaving resources are allocated to patients based on:

- the order in which they arrive
- the seriousness of illness
- what saves the most lives
- how important the patient is to society

[Policy] The allocation policy the triage team decided on is a good one.

- Strongly disagree
- Disagree
- Somewhat disagree
- Neither agree nor disagree
- Somewhat agree
- Agree
- Strongly agree

[Hospital] I would choose this hospital over others if I or someone I loved needed lifesaving treatment.

- Strongly disagree
- Disagree
- Somewhat disagree
- Neither agree nor disagree
- Somewhat agree
- Agree
- Strongly agree

<u>Experiment 2 [Fallo: allocation=0, reallocation=1; Ftype: 0=order, 1=serious, 2=lives, 3=important]</u>

[Allocate – Order] A pandemic is impacting many countries. People infected could die without immediate medical care. The amount of infected people is growing and might soon exceed the capacity of hospitals. The triage team at County General Hospital is responsible for deciding the order that new patients receive lifesaving resources, such as ventilators or ICU beds. The team has recently instituted a new policy. According to this policy, patients will receive lifesaving resources in the order in which they arrive, with those arriving to the hospital first being prioritized. County General is not well supplied, so there are many more patients than there are lifesaving resources available. Because of this, many patients will go without lifesaving treatment who need them.

[Allocate – Serious] A pandemic is impacting many countries. People infected could die without immediate medical care. The amount of infected people is growing and might soon exceed the capacity of hospitals. The triage team at County General Hospital is responsible for deciding the order that new patients receive lifesaving resources, such as ventilators or ICU beds. The team has recently instituted a new policy. According to this policy, patients will receive lifesaving resources in the order of the seriousness of illness, with those who are the worst off being prioritized. County General is not well supplied, so there are many more patients than there are lifesaving resources available. Because of this, many patients will go without lifesaving treatment who need them.

[Allocate – Lives] A pandemic is impacting many countries. People infected could die without immediate medical care. The amount of infected people is growing and might soon exceed the capacity of hospitals. The triage team at County General Hospital is responsible for deciding the order that new patients receive lifesaving resources, such as ventilators or ICU beds. The team has recently instituted a new policy. According to this policy, patients will receive lifesaving resources in the order that saves the most lives, with those having the best chances of recovery being prioritized. County General is not well supplied, so there are many more patients than there are lifesaving resources available. Because of this, many patients will go without lifesaving treatment who need them.

[Allocate – Important] A pandemic is impacting many countries. People infected could die without immediate medical care. The amount of infected people is growing and might soon exceed the capacity of hospitals. The triage team at County General Hospital is responsible for deciding the order that new patients receive lifesaving resources, such as ventilators or ICU beds. The team has recently instituted a new policy. According to this policy, patients will receive lifesaving resources in the order of their importance, with those standing to contribute the most to society being prioritized. County General is not well supplied, so there are many more patients than there are lifesaving resources available. Because of this, many patients will go without lifesaving treatment who need them.

[CC1] There are enough lifesaving resources for every patient who needs them.
- o Yes
- o No

[CC2 – Allocate] Lifesaving resources are allocated to new patients based on:
- o the order in which they arrive
- o the seriousness of illness
- o what saves the most lives
- o how important the patient is to society

[Policy] The policy the triage team decided on is a good one.
- o Strongly disagree
- o Disagree
- o Somewhat disagree

- Neither agree nor disagree
- Somewhat agree
- Agree
- Strongly agree

[Hospital] I would choose this hospital over others if I or someone I loved needed lifesaving treatment.

- Strongly disagree
- Disagree
- Somewhat disagree
- Neither agree nor disagree
- Somewhat agree
- Agree
- Strongly agree

[Reallocate – Order] A pandemic is impacting many countries. People infected could die without immediate medical care. The amount of infected people is growing and might soon exceed the capacity of hospitals. The triage team at County General Hospital is responsible for reallocating lifesaving resources between existing patients, such as ventilators or ICU beds. The team has recently instituted a new policy. According to this policy, lifesaving resources will never be taken from one patient and given to another patient for any reason aside from the patient recovering or expiring. County General is not well supplied, so there are many more patients than there are lifesaving resources available. Because of this, many patients will go without lifesaving resources who need them.

[Reallocate – Serious] A pandemic is impacting many countries. People infected could die without immediate medical care. The amount of infected people is growing and might soon exceed the capacity of hospitals. The triage team at County General Hospital is responsible for reallocating lifesaving resources between existing patients, such as ventilators or ICU beds. The team has recently instituted a new policy. According to this policy, lifesaving resources will be taken from one patient and given to another patient depending on the seriousness of illness, with those who are the worst off receiving priority. County General is not well supplied, so there are many more patients than there are lifesaving resources available. Because of this, many patients will go without lifesaving resources who need them.

[Reallocate – Lives] A pandemic is impacting many countries. People infected could die without immediate medical care. The amount of infected people is growing and might soon exceed the capacity of hospitals. The triage team at County General Hospital is responsible for reallocating lifesaving resources between existing patients, such as ventilators or ICU beds. The team has recently instituted a new policy. According to this policy, lifesaving resources will be taken from one patient and given to another patient depending on what saves the most lives, with those having the best chances of recovery receiving priority. County General is not well supplied, so

there are many more patients than there are lifesaving resources available. Because of this, many patients will go without lifesaving resources who need them.

[Reallocate - Important] A pandemic is impacting many countries. People infected could die without immediate medical care. The amount of infected people is growing and might soon exceed the capacity of hospitals. The triage team at County General Hospital is responsible for reallocating lifesaving resources between existing patients, such as ventilators or ICU beds. The team has recently instituted a new policy. According to this policy, lifesaving resources will be taken from one patient and given to another patient depending on their importance, with those standing to contribute the most to society receiving priority. County General is not well supplied, so there are many more patients than there are lifesaving resources available. Because of this, many patients will go without lifesaving resources who need them.

[CC1] There are enough lifesaving resources for every patient who needs them.

- Yes
- No

[CC2 – Reallocate] Lifesaving resources are reallocated between existing patients based on:

- the patient recovering or expiring
- the seriousness of illness
- what saves the most lives
- how important the patient is to society

[Policy] The policy the triage team decided on is a good one.

- Strongly disagree
- Disagree
- Somewhat disagree
- Neither agree nor disagree
- Somewhat agree
- Agree
- Strongly agree

[Hospital] I would choose this hospital over others if I or someone I loved needed lifesaving treatment.

- Strongly disagree
- Disagree
- Somewhat disagree
- Neither agree nor disagree
- Somewhat agree
- Agree
- Strongly agree

Experiment 3 [Ffair: utilitarian=0, equitable=1; Fgroup: 0=race, 1=disability, 2=elderly, 3=addiction]

[Utilitarian - Race] A pandemic is impacting many countries. People infected could die without immediate medical care. The amount of infected people is growing and has exceeded the capacity of many hospitals. The triage team at County General Hospital is responsible for deciding the order that new patients receive lifesaving resources, such as ventilators or ICU beds. Their current policy aims to save the most lives, with patients having the best chances of recovery receiving priority. The triage team discovers that due to prior risk factors, people of color have a lower chance of recovering from the infection and are thus not receiving the same priority as others for lifesaving resources. The triage team commissions a study into the causes of prior risk factors. But they do not change their policy so that people of color receive the same priority as others. As a result of this, fewer people of color will recover from the infection but more lives will be saved in total.

[Utilitarian - Disability] A pandemic is impacting many countries. People infected could die without immediate medical care. The amount of infected people is growing and has exceeded the capacity of many hospitals. The triage team at County General Hospital is responsible for deciding the order that new patients receive lifesaving resources, such as ventilators or ICU beds. Their current policy aims to save the most lives, with patients having the best chances of recovery receiving priority. The triage team discovers that due to prior risk factors, people with disabilities have a lower chance of recovering from the infection and are thus not receiving the same priority as others for lifesaving resources. The triage team commissions a study into the causes of prior risk factors. But they do not change their policy so that people with disabilities receive the same priority as others. As a result of this, fewer people with disabilities will recover from the infection but more people will recover in total.

[Utilitarian - Elderly] A pandemic is impacting many countries. People infected could die without immediate medical care. The amount of infected people is growing and has exceeded the capacity of many hospitals. The triage team at County General Hospital is responsible for deciding the order that new patients receive lifesaving resources, such as ventilators or ICU beds. Their current policy aims to save the most lives, with patients having the best chances of recovery receiving priority. The triage team discovers that due to prior risk factors, elderly people have a lower chance of recovering from the infection and are thus not receiving the same priority as others for lifesaving resources. The triage team commissions a study into the causes of prior risk factors. But they do not change their policy so that elderly people receive the same priority as others. As a result of this, fewer elderly people will recover from the infection but more people will recover in total.

[Utilitarian - Addiction] A pandemic is impacting many countries. People infected could die without immediate medical care. The amount of infected people is growing and has exceeded the capacity of many hospitals. The triage team at County General Hospital is responsible for deciding the order that new patients receive lifesaving resources, such as ventilators or ICU beds. Their current policy aims to save the most lives, with patients having the best chances of

recovery receiving priority. The triage team discovers that due to prior risk factors, people with substance abuse problems have a lower chance of recovering from the infection and are thus not receiving the same priority as others for lifesaving resources. The triage team commissions a study into the causes of prior risk factors. But they do not change their policy so that people with substance abuse problems receive the same priority as others. As a result of this, fewer people with substance abuse problems will recover from the infection but more people will recover in total.

[Equitable - Race] A pandemic is impacting many countries. People infected could die without immediate medical care. The amount of infected people is growing and has exceeded the capacity of many hospitals. The triage team at County General Hospital is responsible for deciding the order that new patients receive lifesaving resources, such as ventilators or ICU beds. Their current policy aims to save the most lives, with patients having the best chances of recovery receiving priority. The triage team discovers that due to prior risk factors, people of color have a lower chance of recovering from the infection and are thus not receiving the same priority as others for lifesaving resources. The triage team commissions a study into the causes of prior risk factors. And they also change their policy so that people of color receive the same priority as others. As a result of this, more people of color will recover from the infection but fewer people will recover in total.

[Equitable - Disability] A pandemic is impacting many countries. People infected could die without immediate medical care. The amount of infected people is growing and has exceeded the capacity of many hospitals. The triage team at County General Hospital is responsible for deciding the order that new patients receive lifesaving resources, such as ventilators or ICU beds. Their current policy aims to save the most lives, with patients having the best chances of recovery receiving priority. The triage team discovers that due to prior risk factors, people with disabilities have a lower chance of recovering from the infection and are thus not receiving the same priority as others for lifesaving resources. The triage team commissions a study into the causes of prior risk factors. And they also change their policy so that people with disabilities receive the same priority as others. As a result of this, more people with disabilities will recover from the infection but fewer people will recover in total.

[Equitable - Elderly] A pandemic is impacting many countries. People infected could die without immediate medical care. The amount of infected people is growing and has exceeded the capacity of many hospitals. The triage team at County General Hospital is responsible for deciding the order that new patients receive lifesaving resources, such as ventilators or ICU beds. Their current policy aims to save the most lives, with patients having the best chances of recovery receiving priority. The triage team discovers that due to prior risk factors, elderly people have a lower chance of recovering from the infection and are thus not receiving the same priority as others for lifesaving resources. The triage team commissions a study into the causes of prior risk factors. And they also change their policy so that elderly people receive the same priority as others. As a result of this, more elderly people will recover from the infection but fewer people will recover in total.

[Equitable - Addiction] A pandemic is impacting many countries. People infected could die without immediate medical care. The amount of infected people is growing and has exceeded the capacity of many hospitals. The triage team at County General Hospital is responsible for deciding the order that new patients receive lifesaving resources, such as ventilators or ICU beds. Their current policy aims to save the most lives, with patients having the best chances of recovery receiving priority. The triage team discovers that due to prior risk factors, people with substance abuse problems have a lower chance of recovering from the infection and are thus not receiving the same priority as others for lifesaving resources. The triage team commissions a study into the causes of prior risk factors. And they also change their policy so that people with substance abuse problems receive the same priority as others. As a result of this, more people with substance abuse problems will recover from the infection but fewer people will recover in total.

[CC1] The triage team changed their policy regarding lifesaving resources.

- Yes
- No

[Policy] The policy the triage team decided on is a good one.

- Strongly disagree
- Disagree
- Somewhat disagree
- Neither agree nor disagree
- Somewhat agree
- Agree
- Strongly agree

[Hospital] I would choose this hospital over others if I or someone I loved needed lifesaving treatment.

- Strongly disagree
- Disagree
- Somewhat disagree
- Neither agree nor disagree
- Somewhat agree
- Agree
- Strongly agree

Demographic Questionnaire

Demographics

[Sex] Please tell us your biological sex.
- o Male
- o Female

[Age] Please tell us your age.

____

[Lang] Is English a native language for you?
- o No
- o Yes

[Race] What is your ethnicity?
- o White
- o Black
- o Hispanic
- o Asian
- o Other

[Edu] What is the highest level of education you have completed?
- o High School
- o Some College
- o 2 Year Degree
- o 4 Year Degree
- o Postgraduate or Professional degree

[Relig] I consider myself a religious person.
- o Strongly disagree
- o Disagree
- o Somewhat disagree
- o Neither agree nor disagree
- o Somewhat agree
- o Agree
- o Strongly agree

[Poly_eco] How economically conservative or liberal are you?
- o Extremely Conservative
- o Conservative
- o Slightly Conservative
- o Moderate

- Slightly Liberal
- Liberal
- Extremely Liberal

[Poly_soc] How socially conservative or liberal are you?
- Extremely Conservative
- Conservative
- Slightly Conservative
- Moderate
- Slightly Liberal
- Liberal
- Extremely Liberal

[Hosp] Have you been admitted to the hospital any time in the last 5 years?
- Yes
- No

[Hprof] Have you ever worked or studied as a health professional (e.g. nurse, doctor, therapist, etc)?
- Yes
- No

[State] In which state do you currently reside?

____
