# Supplementary material for: Public attitudes toward allocating scarce resources in the COVID-19 pandemic
Source: PLoS One. 2020 Nov 4;15(11):e0240651. doi: 10.1371/journal.pone.0240651 (PMC7641460; doi:10.1371/journal.pone.0240651)
Supplement: Attachment — Submitted filename: Response to Reviewers PLOS ONE.docx [file pone.0240651.s003.docx]

Authors’ Report

“Public Attitudes Toward Allocating Scarce Resources in the COVID-19 Pandemic”

*PLOS ONE*

September 26, 2020

We are grateful for the opportunity to submit a revision of this manuscript to *PLOS ONE.* We are pleased that the reviewers’ assessed the manuscript as an “extremely well-presented paper” that represents a “timely and important contribution” to the literature. We also appreciate that Reviewer 2 not only raised several insightful criticisms, but also offered concrete suggestions to address them. In the following report, we review the main points raised by Reviewer 2 and explain how we have responded to them. We have instituted the suggested changes in all cases, and, in our judgment, this has significantly improved the manuscript.

First, Reviewer 2 writes that the wording used to describe the lack of support for policies in the abstract and discussion could be misleading in cases where participants are ambivalent and, relatedly, that participant resistance to utilitarian reallocation is overstated. We agree with Reviewer 2 and have made revisions throughout the manuscript to avoid this. In the abstract (p. 2) and experimental discussion sections (pp. 13, 20, 26) we have revised the language regarding interpretations of participant ambivalence. We also call attention to the fact that resistance to utilitarian reallocation is not as strong as one might have predicted given the controversial nature of withdrawing life-sustaining therapy (p. 30).

Related to this point, the reviewer also notes that the phrase “abandoning utilitarian allocation” could be misleading because participants only expressed views about one variation in allocation principles. Reviewer 2 suggests that this be noted as a limitation. In the revised manuscript, we remove this phrase and note more explicitly this fact as a limitation (p. 32).

Second, Reviewer 2 objects to our use of the term “prioritarian” to describe a policy that prioritizes patients who are the worst-off in terms of the severity of illnesses. Reviewer 2 reasons that: (i) there are other ways that patients might be considered worst-off, and (ii) participants might have ultimately been thinking about allocation on utilitarian grounds rather than prioritarian grounds. Review 2 also makes a similar, but minor, point regarding our presentation of the maximizing benefits principle, where maximizing benefits could be interpreted as saving the most lives, the most life years, or a combination of both.

We agree that participants may have had various interpretations of these ethical principles and that these interpretations may not reflect the way some theorists classify prioritarian or utilitarian theories. To address these concerns, we make several revisions to the manuscript:

- We remove the term “prioritarianism” from the manuscript as Reviewer 2 suggests. We replace this term with variations of “worst-off” or “seriousness of illness” that better align with the language used in our experimental vignettes.
- We re-write the paragraph(s) in the introduction introducing ethical principles (p. 3). This now makes it clear that (i) ethical principles are used to motivate specific triage policies, (ii) triage policies motivated by these principles might be instituted in a number of different ways, and (iii) there is scholarly debate about which triage policies best capture underlying allocation principles in practice.
- We expand the limitations section to explicitly discuss disagreements about ethical principles, using utilitarianism as an example (p. 32). We note that there is an important theoretical disagreement concerning which policy should rightfully be called “utilitarian” as well as discuss important experimental reasons to question whether underlying psychological processes associated with “utilitarian” answers truly reflect utilitarian reasoning.
- We add a paragraph to the general discussion exploring the hypothesis that participants interpreted allocation based on severity of illness as justified by maximizing benefits (p. 31). Though this could be a natural interpretation, interestingly, to the extent that participants evaluate these policies differently, there is some evidence to suggest that they were not processing the cases in that way.

Third, Reviewer 2 writes that “As a limitation, it would be important to acknowledge that these surveys were conducted with US citizens and may not be applicable to other countries”. We have added this to the list of study limitations on p. 31.

Fourth, Reviewer 2 writes that the description of prior work by Huang and colleagues may be misleading because veil of ignorance manipulations are not usually taken to prime utilitarian thinking. We correct this description on p. 29.

Fifth, Reviewer 2 asks why dependent variables were combined for analysis. In the revised manuscript we clarify that this combination was specified in our preregistration document to a create a scale if responses to different conditions were extremely similar because items were tapping into the same underlying construct. However, the data are freely available through the Open Science Framework if others wish to explore the data along these dimensions separately. Along with this, we also remove the phrase “categorically refusing to attend hospitals” singled out by the reviewer.
